# Physical activity of older children and adolescents in Germany – Results of the HBSC study 2022 and trends since 2009/10

**DOI:** 10.25646/11874

**Published:** 2024-03-04

**Authors:** Jens Bucksch, Juliane Möckel, Anne Kaman, Gorden Sudeck, for the HBSC Study Group Germany

**Affiliations:** 1 Heidelberg University of Education, Faculty of Natural and Social Sciences, Department of Prevention and Health Promotion; 2 Heidelberg University of Education, Heidelberg Centre for Prevention and Health Promotion; 3 University Medical Centre Hamburg-Eppendorf, Department of Child and Adolescent Psychiatry and Psychotherapy, Child Public Health Research Section; 4 Eberhard Karls University of Tübingen, Institute of Sports Science; 5 Eberhard Karls University of Tübingen, Interfaculty Research Institute for Sport and Physical Activity

**Keywords:** CHILDREN, ADOLESCENTS, PHYSICAL ACTIVITY RECOMMENDATION, PHYSICAL ACTIVITY, SPORTING ACTIVITY, SCHOOLS, HBSC, SURVEY, PREVALENCE, GERMANY

## Abstract

**Background:**

Physical activity is central to health, beginning in childhood and adolescence, and regular monitoring provides important information for strategic decisions on promoting physical activity in Germany.

**Methods:**

The current survey cycle of the Health Behaviour in School-aged Children (HBSC) study gives an insight into the prevalence of the indicators daily recommended physical activity, high and low physical activity, and sporting activity among students aged between 11 and 15 for 2022. In addition, the data is compared to the survey cycles of the 2009/10, 2013/14, and 2017/18 school years and analysed over time.

**Results:**

The results of the current survey cycle show that 10.8 % of girls, 20.9 % of boys, and 12.4 % of gender diverse adolescents fulfil the daily physical activity recommendation. There are also major gender-specific differences for the other indicators. The group of gender diverse adolescents needs to be analysed further. The changes over time between 2009/10 and 2022 are relatively small. While girls’ physical activity habits decreased slightly for the various indicators between 2009/10 and 2022, boys’ prevalence remained relatively stable over the same period.

**Conclusions:**

Overall, in part due to the effects of the various COVID-19 lockdowns, the need for effective and population-based measures to promote physical activity in childhood and adolescence remains high.

## 1. Introduction

Physical activity is a key influencing factor for health across all phases of life. Even children’s and adolescents’ physical and mental health benefits from physical and sporting activities [1, 2]. In addition, the preventive effect of physical activity on various chronic, non-communicable diseases (e.g. type II diabetes, heart attack, bowel cancer) in adulthood has been strongly proven [3]. If health-promoting physical activities are socially and ritually established in children and adolescents at an early age, active children are more likely to become active adults [4, 5].

According to the updated international recommendations of the World Health Organization (WHO), adolescents should be physically active for at least 60 minutes a day with at least moderate intensity (e.g. cycling at 15 km/h). This activity can be reached in everyday life or during leisure time. In the recently updated version of the WHO physical activity recommendation at international level, the focus for children and adolescents has shifted from daily physical activity time to the total weekly amount of at least moderate-intensity physical activity. There is sufficient evidence in favour of a weekly average of 60 minutes per day causing positive health effects rather than a daily target of 60 minutes. In addition, it is pointed out that children and adolescents should perform aerobic-orientated and muscle-strengthening activities with higher-intensity use of large muscle groups at least three days a week in order to achieve further effects for healthy development. High-intensity activities generally involve activities that significantly increase the heart rate. In addition, the time spent sitting should be limited [6].

Although the health potential is undisputed, many children and adolescents globally and nationally fall short of the physical activity recommendation. According to a comparative international analysis of available data, 22.4 % of 11 to 17-year-old boys and 15.3 % of girls of the same age worldwide fulfil the WHO recommendation for daily physical activity of at least 60 minutes. At 20.3 % and 12.1 %, respectively, the figures for Germany are below the global average in this comparison. The data stem from a compilation of 298 population-based surveys in this age group and were collected by means of adolescents’ self-reporting [7]. In addition, the Active Healthy Kids Global Alliance issues physical activity report cards in the form of the Global Matrix for 57 countries based on various indicators of physical activities, which summarise existing studies such as the Health Behaviour in School-aged Children (HBSC) study in a secondary analysis. In the latest Global Matrix 4.0, ‘weak’ grades or a low level of physical activity are found across the board, with Germany scoring ‘D-’ in this regard [8]. If, in comparison, physical activity is measured using objective, device-based measurement methods (e.g. accelerometry), even fewer children and adolescents fulfil the health-oriented physical activity recommendation [9].

Another clear finding at both international and national level is that, on average, girls are less (physically) active than boys [9]. On average, girls also lose more time spent with physical activity than boys from childhood to adolescence [10]. If sporting activity is considered, there are recurring clear differences between the genders, both internationally and nationally, at the expense of girls and at the expense of girls and boys from socioeconomically disadvantaged families [11].

The extent to which the physical activity of children and adolescents have changed in recent decades is the subject of frequent scientific debate. A systematic review based on international and national data shows roughly the same number of studies reporting an increase or decrease in physical activity between 1970 and 2018 [12]. In addition, the Global Matrices 1.0 to 4.0 evaluate the current situation regarding physical activity and sporting activities at a low grade in various countries over time. Across the participating countries, positive developments can only be seen in free play and active transport (e.g. walking or cycling to school). It is again striking that these positive developments are less evident in girls than in boys [13]. For the German national context, the assessment of physical activity using the Global Matrices remains at a low level between 2018 and 2022 [8, 14].

For Germany, the latest cycles of the HBSC study between 2009/10 and 2017/18 showed that both the fulfilment of the physical activity recommendation for total physical activity per day and specifically sporting activity are slightly declining – for both girls and boys. At the same time, the proportion of girls and boys with low levels of physical activity increased over time [11]. Further analyses between the 2001/02 and 2013/14 survey dates of the HBSC study report relatively stable values for overall physical activity and sporting activity in particular [15]. For longer-term trends of physical activity, it should be emphasised that the prevalence data refer to the period before the COVID-19 pandemic and related lockdowns, which were associated with significant effects on health and health behaviour for children and adolescents [16]. Studies on physical activity conducted during the COVID-19 pandemic and related lockdowns observed a decline in the prevalence of physical activity [17, 18]. The magnitude of the decline is estimated at - 10.8 min/day to - 91 min/day, with the greatest losses being observed in the area of structured activities and organised sport. Boys were less affected by the reduction [19].

Against this background, the aim of this article is to use the current data from the HBSC study 2022 to present the prevalence of various indicators of physical activity in late childhood and adolescence. With reference to the COVID-19 pandemic and the associated restrictions, it should also be noted that the current HBSC data were collected at a time when physical and sporting activities in informal and organised forms were largely permitted again. In addition, temporal trends since the 2009/10 HBSC survey cycle are reported, allowing the development of physical activity to be tracked at four-year intervals on the basis of the nationwide surveys.

**HBSC 2022**

**Data holder:** HBSC Study Group Germany

**Objective:** The aim of the study is to analyse the health and health behaviour of students. Continuous health monitoring through the HBSC study contributes to informing decision-makers in policy and practice about the current fields in prevention and health promotion in childhood and adolescence. A particular focus is on the influencing factors and the social contexts of health in the young generation.

**Study design:** Cross-sectional survey by written questionnaire every four years

**Population:** Students with average ages 11, 13, and 15

**Sampling:** Observation units are schools and the class groups clustered within them. From the population of all state general education schools in Germany, a cluster sample was drawn. In order to obtain a representative estimate (close to the distribution of the population), school size and the percentage distribution of students were included in the sampling, stratified by school type and federal state (Probability Proportional to Size (PPS) design).

**Data collection period:** March – November 2022

**Sample size:**

**2022:** 6,475 students

**All four survey cycles (2009/10-2022):** 21,788 students

**HBSC survey cycles:**

**Included in the articles in this issue of the Journal of Health Monitoring:**

▶ 2009/10
▶ 2013/14
▶ 2017/18
▶ 2022

More information can be found at https://hbsc-germany.de/ (German)

## 2. Methods

### 2.1 Sample design and study implementation

The Health Behaviour in School-aged Children (HBSC) study is designed as a cross-sectional study that takes place every four years in a school setting and surveys students aged around 11, 13 and 15 (mean deviation of 0.5 years). In Germany, these age groups mainly comprise grades 5, 7, and 9. Students at general education schools in all 16 federal states in Germany have been surveyed in the school years 2009/10, 2013/14, 2017/18 and in the calendar year 2022 as part of the HBSC study. The schools approached for participation were drawn as a cluster sample from the population of all state general education schools in Germany. In order to obtain a representative estimate (close to the distribution of the population), school size and the percentage distribution of students were included in the sampling, stratified by school type (Probability Proportional to Size (PPS) design).

The HBSC study is conducted by means of a questionnaire, which the students complete themselves. The study has been approved by the responsible ministries or state education authorities in all federal states (except North Rhine-Westphalia, as the decision of participation lies within the schools in this federal state).

Four survey cycles of the HBSC study Germany were analysed for the present study. In addition to the current survey in 2022 (n = 6,475), three further surveys were included in the following school years: 2009/10 (n = 5,005), 2013/14 (n = 5,961), and 2017/18 (n = 4,347). All data sets were standardised and adjusted by the international HBSC consortium so that the age groups are comparable. Further information on the HBSC study and the methodology can be found in the article by Winter & Moor [20] in this issue of the Journal of Health Monitoring.

### 2.2 Survey procedure

This article focuses on indicators for physical activity as well as gender and age as sociodemographic stratification variables. Gender was recorded in the 2022 survey year using the three options ‘girl’, ‘boy’, or ‘diverse’. In the previous survey cycles, gender was recorded in binary form (girl, boy). For the trend analyses, people who did not specify their gender or classified themselves as diverse were excluded from the gender-specific analyses. The age was determined at the time of the survey using the information provided by the students on their month and year of birth and summarised with a deviation of +/- 0.5 years into the age categories ‘11 years’, ‘13 years’ and ‘15 years’. For regression analyses, socioeconomic status is also included as a control variable for the 2022 survey cycle. This is determined in the HBSC study using the Family Affluence Scale (FAS) to measure the social status of adolescents. Six items (car, own room, vacations taken with the family, computer, number of bathrooms, dishwasher) are added and categorised as low, medium, or high family affluence based on the percentage distributions within the sample [21].

#### Indicators of physical and sporting activities

Physical activity of at least moderate intensity was determined by asking on how many of the last seven days the older children and adolescents had been physically active for at least 60 minutes. The introduction to this item uses examples to illustrate that it refers to all physical activities throughout the day that elevate the pulse rate and cause you to get out of breath for a while. The respondents ticked one of the eight response categories from zero to seven days. Three indicators were formed on the basis of these response categories. Firstly, it was determined whether the respondents were engaged in 60 minutes of moderate-intensity physical activity per day and thus fulfil the health-effective level of physical activity according to the minimum duration per day (‘physical activity recommendation fulfilled’). The survey instrument thus still refers to the WHO recommendation of at least 60 minutes per day, although the update of the recommendations introduced an average daily target of 60 minutes, as described above [6]. The criterion was chosen as an approximation of the updated physical activity recommendation to allow comparisons of HBSC cycles over time. We also use other indicators to further characterise physical activity. On the one hand, adolescents with ‘low physical activity’ who engage in 60 minutes of moderate-intensity physical activity on between zero and two days are identified, as well as those adolescents with ‘high physical activity’ who were physically active for at least 60 minutes of moderate-intensity physical activity on five or more days. This approach can also be found in other publications of the HBSC network [22].

In addition to information on general physical activity, sporting activities are also surveyed in the HBSC studies. In principle, sporting activities are already included in the first indicator, as the information relates to all activities in leisure time and everyday life. The exact intensity can therefore not be determined from this item and various studies emphasise that the proportion of sporting activities contribute to overall physical activity as one dimension among others [23]. However, in order to enable a separate consideration of sporting activities with their often higher intensities, the participants were asked how often they exercise in their free time causing them to get out of breath or break into a sweat. The respondents chose from seven possible answers, ranging from ‘every day’ to ‘never’. For the analyses, the answers were dichotomised in line with international reports from the HBSC study with the reference variable ‘sporting activities on at least four days a week’ [24].

### 2.3 Statistical methods

The main results are described separately for girls, boys, and gender diverse adolescents as prevalences or percentage frequencies with a 95 % confidence interval (CI), with additional stratification by age. Deviations in the number of respondents between the indicators of physical activity are due to the different number of missing values. Binary logistic regressions were calculated to statistically substantiate the descriptive information in the comparison of different subgroups of the sample. The relationship between the sociodemographic characteristics of gender, age, and family affluence and the various indicators is estimated using regression models with adjustment for all other included variables. The results are presented as odds ratios (OR) and 95 % CI. The temporal trends of the characteristics of physical activity habits are described for the last four survey cycles using the gender-specific percentage frequencies. In addition, binary logistic regressions were calculated separately for girls and boys, by using various survey cycles- in dummy format as independent variables (reference category: survey cycle 2009/10) and age as a control variable. In this way, changes in the indicators of physical activity habits over time are statistically substantiated using OR and 95 % CI. In addition, the trend was tested for linearity by treating the survey cycles as a categorical variable in the regression analyses controlled for age. All analyses were performed with SPSS 28. The significance level is set at p < 0.05 for all inferential statistical methods.

A weighting factor was created for all survey cycles to ensure nationwide sample representativeness. This equalises different participation rates in the federal states and school types so that the distribution corresponds to the population. Due to the weighting, all three age categories and the binary gender categories of girls and boys are included in the analyses in equal parts from the 2017/18 survey cycle onwards. In the 2022 HBSC survey cycle, gender was not recorded exclusively in binary form for the first time, with 1.7 % of respondents indicating the category gender diverse. This was taken into account in the weighting of the 2022 data, while girls and boys were weighted equally (49.2 % each; participants who did not specify their gender were excluded). Further details on the weighting of the data can be found in the article by Winter & Moor [20].

## 3. Results

###  

#### Physical and sporting activity indicators for the 2022 survey cycle

The physical activity recommendation is fulfilled by 10.8 % of girls, 20.9 % of boys, and 12.4 % of gender diverse adolescents. In all gender categories, the proportion fulfilling this requirement decreases in the older age groups (Table 1). The logistic regression analyses underline a statistically significant difference between the gender categories and the age groups (Table 2). The lowest values were found among 15-year-old gender diverse adolescents, only 4.5 % of whom reported being physically active for at least 60 minutes a day. The highest values were found among 11-year-old boys (26.5 %). The results of the logistic regression are controlled for family affluence and confirm the correlations with the age and gender categories shown in the descriptive data.

For the indicator of a high level of physical activity, which is defined as 60 minutes of moderate-intensity physical activity on five to seven days per week, the situation is similar across the gender and age categories with significantly higher prevalences, which correspond to around two to three times the ‘physical activity recommendations fulfilled’ indicator (Table 1); however, there are only slight differences between 13- and 15-year-old girls and boys for the indicator of a high level of physical activity. The highest value is again found among 11-year-old boys (49.6 %), while the 15-year-old gender diverse adolescents have the lowest value (23.6 %).

Looking at the category with low physical activity, it can be seen that a fifth of boys and a third of girls are physically active for 60 minutes on less than three days a week (Table 1). Here, too, the gender difference is statistically significant (Table 2). It is particularly noticeable that gender diverse adolescents have by far the highest prevalence of low physical activity (every second adolescent in this group). Taking into account the age categories, there is also an increasing proportion of low physical activity among girls from the 11-year-olds to 13-year-olds to 15-year-olds, while no age differences can be observed among boys (Table 1). In the regression model, the correlations visible in the descriptive data are confirmed when controlling for family affluence (Table 2).

There are also strong differences between the gender categories when it comes to sporting activity on at least four days a week. Almost half of boys (49.8 %) are active in sport at least four days a week, whereas the proportions of girls and gender diverse adolescents are 28.2 % and 19.1 %, respectively. In the latter two gender categories, there are also strong correlations with age. For girls, the proportion of sporting activities at more than four days per week decreases by half from 11-year-olds to 15-year-olds. For boys, on the other hand, changes can be observed from 11-year-olds to 13-year-olds, although these are smaller overall. The regression model reflects the descriptive data regardless of family affluence (Table 2).

#### Indicators of physical and sporting activity over time

**Figure 1:**
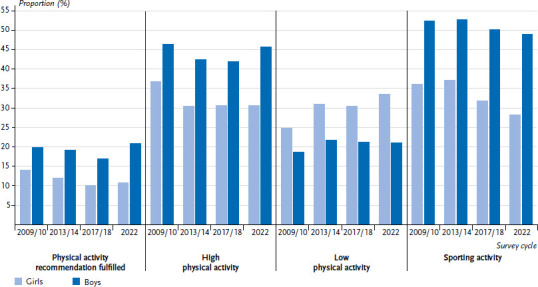
Fulfilling the physical activity recommendation, physical and sporting activity by gender and HBSC survey cycle (2009/10: n = 2,525 girls, n = 2,364 boys, 2013/14: n = 2,857 girls, n = 2,967 boys, 2017/18: n = 2,278 girls, n = 2,021 boys, and 2022: n = 3,258 girls, n = 3,074 boys) Source: HBSC Germany 2009/10, 2013/14, 2017/18, 2022

Figure 1 shows the prevalence of the four physical activity indicators over the last four HBSC survey cycles. If the data is compared chronologically in ascending order from 2009/10 onwards, the daily physical activity recommendation is fulfilled less frequently by girls over time, with a slight increase from 2017/18 to 2022. The difference between 2009/10 and 2022 is three percentage points. For boys, the proportion remains relatively stable over time with the highest prevalence in 2022.

The other indicators of physical activity complete the picture. While the proportion of boys with low physical activity, i.e. at least 60 minutes of moderate-intensity activity on zero to two days per week, increased by around three percentage points after 2009/10 and remained at this level in the subsequent survey cycles, girls saw an increase of 8.7 percentage points from 2009/10 to 2022.

For the indicator of a high level of physical activity, i.e. at least 60 minutes of moderate-intensity activity on five to seven days per week, a decrease of 6.1 percentage points can be seen among girls for the period mentioned. For boys, this difference is 0.6 percentage points, with the percentages for the 2013/14 and 2017/18 survey cycles being slightly lower.

Finally, a decline in sporting activity over time can be seen for both girls and boys. The differences are somewhat more pronounced for girls. The lowest proportion of 11- to 15-year-olds who are active in sport at least four times a week is 38.6 % of girls in the 2022 survey cycle. The reduction is particularly noticeable among girls from 2013/14 to 2017/18.

Regression analyses on these changes over time were carried out, statistically confirmed for the age effect and are presented in Table 3 and Table 4 with the reference category 2009/10.

For girls, it is statistically significant that there was a decrease in physical and sporting activities and an increase in low physical activity between 2009/10 and 2022. The trend effects over time are comparably high for the various indicators. A closer look reveals the following statistically significant differences with the 2009/10 reference category:

▶ for comparison with 2017/18 for achieving the physical activity recommendation,
▶ for the comparison with the 2022 survey cycle for low physical activity and a decrease in sporting activities (Table 3).

For boys, it is clear that a significant decrease can be observed for sporting activities over the period from 2009/10 to 2022. No significant trend can be identified for the other indicators of physical activity. There is a statistically significant reduction in the proportion of boys fulfilling the physical activity recommendation and high levels of physical activity compared to 2017/18 and 2009/10 (Table 4).

## 4. Discussion

In this article, the HBSC data from the 2022 survey cycle were analysed with regard to physical activity based on self-reports by 11-, 13-, and 15-year-old children and adolescents on general physical activity and sporting activities. Gender-specific differences and differences between the three age categories are striking: On average, girls show less physical and sporting activities than boys; with increasing age, girls and boys exhibit less physical and sporting activities. There are also indications that gender diverse adolescents have very low levels of physical and sporting activities. In summary, the data on the time trends for fulfilling the physical activity recommendation and a high level of physical activity show that the changes between 2009/10 and 2022 are relatively small.

In view of the observation that only one tenth of girls and one fifth of boys achieve the recommended 60 minutes of physical activity per day, a frequent lack of physical activity must be stated in the age groups between 11 and 15 years. This has been exacerbated over time for girls. It should therefore be noted that the need for effective and population-based measures to promote physical activity in children and adolescents remains high and that previous efforts have not yet been able to reverse the trend. The authors come to a similar conclusion based on the Global Matrices for both the national and international context [8, 13, 14]. Our findings are confirmed by further studies [7, 25].

In our data, it is also striking that the proportion of children and adolescents with significantly too little physical activity has increased substantially among girls. The trend for boys is not as clear as for girls and shows stable prevalences, which nevertheless continue to signal a high need for intervention. Other studies confirm that the extent of low physical activity affects a non-negligible proportion of the adolescent population and see an overall increase in this proportion among adolescents [25, 26].

With regard to the frequency of sporting activities, the current data from the HBSC study confirms the findings of quite high prevalences and an imbalance in favour of boys [15, 27, 28]. Based on the Global Matrix, the general conditions for organised sport and for playing sport in clubs are assessed as comparatively positive in Germany [8, 14]. In the MoMo studies (motor skills module of the German Health Interview and Examination Survey for Children and Adolescents, KiGGS), sporting activities were surveyed in a more differentiated way, with the data showing clear differences in adolescence between girls and boys, particularly for extracurricular sport, which takes place in sports clubs or in informal contexts [29]. With regard to the temporal trends of sporting activities, a significant decrease can be observed for both genders, which is more pronounced for girls. This development is a cause for concern, as it further widens the gap between girls and boys in terms of participation in sports. However, the data from the MoMo study found comparable prevalences between the two survey cycles 2003 to 2006 and 2014 to 2017 and found no significant differences over time [29]. The measurement methodology in the MoMo study is more differentiated and the age range is not comparable with the sample of our study.

The comparative studies presented so far relate to the situation before the COVID-19 pandemic. There are indications that the amount of time children and adolescents spent with physical activity has decreased due to the pandemic [17, 18], particularly in structured activities and organised sport [19]. This conclusion is also consistent with our own findings, as we see a temporal trend towards less sporting activities over time in both girls and boys, with the lowest values being achieved in the current cycle. Other studies also show that the lockdown in the winter months of January/February 2021 led to significantly greater losses in physical activity habits compared to the lockdown in spring 2020. Organised sport activities play a greater role in the winter months in particular [30, 31]. However, boys appear to be less affected by the reduction [19]. This could be an indication that the prevalence of sporting activities in our data for girls is even less favourable over time compared to boys. It should also be pointed out again that our data collection took place in 2022 and reflects a situation in which many sporting activities could already be resumed in an informal and formal context. Overall, various studies indicate that the amount of physical activity has not yet fully recovered after the COVID-19 lockdown and corresponds roughly to an additional loss that is expected to occur during adolescence anyway [32].

In addition to the clear difference in prevalence between girls and boys, there are remarkable results for gender diverse adolescents. In the current survey cycle, it was possible to categorise oneself as ‘girl’, ‘boy’, or ‘diverse’. It was found that the physical and sporting activities of gender diverse adolescents was significantly lower than that of girls and boys. In the group of 15-year-old gender diverse adolescents in particular, only 4.5 % fulfilled the physical activity recommendation, around 50 % were physically active for at least 60 minutes on less than three days a week and only 11 % were physically active at least four times a week. Overall, this issue has rarely been investigated. The Minnesota Student Survey confirmed large differences in organised sports and general physical activity between those who classified themselves as boys or girls vs. those who classified themselves as transgender, genderqueer, genderfluid, or unsure about their gender identity [33]. However, the findings on the differences between girls and boys on the one hand and gender diverse adolescents on the other should be put into perspective, as the variance explanation within the logistic regression models is low, meaning that other influencing factors in addition to sociodemographic factors (e.g. living environment, motivational mindset) are significant.

Our results should also be categorised against the background of some limitations. Firstly, all the data from the HBSC study are self-reported questionnaire responses from children and adolescents, which are subject to memory bias compared to device-based, objective measurement methods. The prevalences should therefore be used more as a benchmark, but are suitable as a measure over time. Even if our measurement instrument probably overestimates the overall level of physical activity, studies in adolescence that measured physical activity using devices (such as accelerometry, pedometers) also confirm a decline over time [34, 35]. In addition, we must emphasise that the screening question on fulfilling the physical activity recommendation does not match the updated physical activity recommendation, as the accumulated physical activity time over the course of the week has become more relevant than the ‘strict’ daily physical activity time of 60 minutes [6, 36]. The development of new reliable and valid survey instruments is of high priority in order to collect adequate and comparable data in the future [37]. With regard to the time trends, it should be added that we only report on cross-sectional trends and do not present longitudinal data on changes in physical activity over time.

In addition to the implications for the gender-sensitive development of physical activity promotion measures [38], the results of the current HBSC survey cycle and the development over time from 2009/10 to 2022 show that there is a great need for interventions. Previous measures have not yet contributed to a sufficient spread of physical and sporting activity in the young population, which significantly reduces the probability of positive transfer effects into adulthood [5]. It is clear from the data that comprehensive promotion of physical activity for older children and adolescents can only be achieved through a combined strategy if, in addition to promoting structured sports programmes in sports clubs and strengthening physical education, it also addresses the general promotion of physical activity in leisure and everyday life. Both play a central role. There is no simple answer to the question of the specific type of intervention required. There is much to be said for an optimised combination of structural and behavioural prevention approaches [39]. For example, various studies show that everyday journeys, e.g. to school, are travelled more actively if there are safe cycle/walking routes and options for stowing heavy and bulky objects, and if motivational aspects are also strengthened at the same time [40, 41]. Successful implementations include the ‘walking school bus’ or the so-called bicycle train [42]. In addition, the community setting appears to be a promising basal approach for the promotion of physical activity, as it subsumes various settings and unites them under one roof and at the same time creates the conditions and framework for physical activity for all people – for example by prioritising walking and cycling [43, 44].

## Key statement

- Only 10.8 % of girls, 20.9 % of boys and 12.4 % of gender diverse adolescents fulfil the WHO recommendation for daily physical activity.
- Boys are more active than girls; this difference is much more pronounced for sporting activity compared to physical activity.
- Physical and sporting activities decrease with age; the differences between girls and boys increase between the ages of 11 and 15.
- Gender diverse young people are less physically active and less involved in sporting activities.
- From 2009/10 to 2022, physical and sporting activities among girls decreased for the various indicators; relatively stable prevalence rates can be observed among boys.

## Figures and Tables

**Table 1:** Fulfilling the physical activity recommendation, physical and sporting activity by gender and age (n = 3,258 girls, n = 3,074 boys, n = 112 gender diverse) Source: HBSC Germany 2022

Age	Physical activity recommendation fulfilled^1^ (n = 6,214)		High physical activity^2^ (n = 6,214)		Low physical activity^3^ (n = 6,214)		Sporting activity (≥ 4 days per week) (n = 6,192)	
	%	(95 % CI)	%	(95 % CI)	%	(95 % CI)	%	(95 % CI)
**Girls**	**10.8**	**(9.4 – 12.3)**	**30.7**	**(28.6 – 32.8)**	**33.6**	**(31.3 – 35.9)**	**28.2**	**(26.3 – 30.2)**
11 years	14.7	(12.3 – 17.6)	36.4	(32.9 – 40.2)	28.6	(25.3 – 32.1)	40.3	(36.6 – 44.1)
13 years	10.5	(7.9 – 13.8)	28.7	(25.1 – 32.6)	32.9	(28.7 – 37.3)	24.3	(21.3 – 27.6)
15 years	7.4	(5.7 – 9.5)	27.1	(23.9 – 30.5)	38.9	(35.0 – 42.9)	20.4	(17.7 – 23.5)
**Boys**	**20.9**	**(19.0 – 23.0)**	**45.7**	**(43.3 – 48.1)**	**21.0**	**(19.2 – 23.0)**	**49.8**	**(46.5 – 51.3)**
11 years	26.5	(23.0 – 30.4)	49.6	(45.7 – 53.5)	21.8	(18.8 – 25.2)	54.1	(50.2 – 58.1)
13 years	17.8	(15.1 – 20.8)	43.8	(39.7 – 48.0)	19.7	(16.8 – 23.0)	46.9	(42.7 – 51.1)
15 years	18.1	(14.8 – 22.1)	43.5	(39.3 – 47.8)	21.6	(18.1 – 25.5)	45.5	(41.2 – 49.8)
**Gender diverse^4^**	**12.4**	**(7.2 – 20.6)**	**23.6**	**(16.3 – 33.0)**	**48.2**	**(37.0 – 59.7)**	**19.1**	**(12.4 – 28.4)**
13 years	21.6	(10.1 – 40.3)	34.9	(20.3 – 53.0)	47.3	(30.2 – 65.0)	26.7	(14.0 – 45.0)
15 years	4.5	(1.8 – 11.2)	23.6	(16.3 – 33.0)	49.9	(34.9 – 65.0)	11.0	(5.8 – 19.8)

CI = confidence interval

^1^ Physical activity recommendation fulfilled = 60 minutes of physical activity seven days a week

^2^ High physical activity = 60 minutes of physical activity on five to seven days a week

^3^ Low physical activity = 60 minutes of physical activity on less than three days per week

^4^ The data on the 11-year-old gender diverse adolescents was not presented, as this group comprises only seven people.

**Table 2:** Fulfilling the physical activity recommendation, physical and sporting activity by gender and age, multivariate logistic regression model with inclusion of all predictors and family affluence Source: HBSC Germany 2022

	Physical activity recommendation fulfilled^1^ (n = 6,072)		High physical activity^2^ (n = 6,072)		Low physical activity^3^ (n = 6,072)		Sporting activity (≥ 4 days per week) (n = 5,776)	
	OR	(95 % CI)	OR	(95 % CI)	OR	(95 % CI)	OR	(95 % CI)
**Age**								
11 years (Ref.)	1		1		1		1	
13 years	0.61	(0.49 – 0.77)***	0.72	(0.61 – 0.86)***	1.10	(0.90 – 1.33)	0.59	(0.49 – 0.70)***
15 years	0.53	(0.41 – 0.67)***	0.68	(0.57 – 0.81)***	1.34	(1.11 – 1.61)***	0.51	(0.43 – 0.61)***
**Gender**								
Boys (Ref.)	1		1		1		1	
Girls	0.44	(0.36 – 0.54)^***^	0.50	(0.44 – 0.58)^***^	1.97	(1.68 – 2.31)^***^	0.39	(0.34 – 0.45)^***^
Gender diverse	0.64	(0.34 – 1.17)	0.38	(0.24 – 0.62)^*^	3.46	(2.10 – 5.69)^***^	0.32	(0.19 – 0.55)^***^
Nagelkerkes R^2^	0.062		0.066		0.062		0.112	

OR = Odds ratio, CI = confidence interval

^*^ significant p < 0.05

^**^ very significant p < 0.01

^***^ highly significant p < 0.001

^1^ Physical activity recommendation fulfilled = 60 minutes of physical activity seven days a week

^2^ High physical activity = 60 minutes of physical activity on five to seven days a week

^3^ Low physical activity = 60 minutes of physical activity on less than three days per week

**Table 3:** Fulfilling the physical activity recommendation, physical and sporting activity of girls, temporal trends (odds ratio, 95 % confidence interval) over the HBSC survey cycles 2009/10 (n = 2,525), 2013/14 (n = 2,857), 2017/18 (n = 2,278), and 2022 (n = 3,258), controlled for age Source: HBSC Germany 2009/10, 2013/14, 2017/18, 2022

Girls	Physical activity recommendation fulfilled^1^ (n = 10,749)		High physical activity^2^ (n = 10,749)		Low physical activity^3^ (n = 10,749)		Sporting activity (≥4 days per week) (n = 10,657)	
	OR	(95 % CI)	OR	(95 % CI)	OR	(95 % CI)	OR	(95 % CI)
**Date of survey**								
2013/14 vs. 2009/10	0.85	(0.72 – 0.99)^*^	0.76	(0.68 – 0.85)^***^	1.36	(1.20 – 1.53)^***^	1.06	(0.95 – 1.19)
2017/18 vs. 2009/10	0.69	(0.58 – 0.83)^***^	0.76	(0.67 – 0.86)^***^	1.32	(1.16 – 1.51)^***^	0.83	(0.73 – 0.94)^**^
2022 vs. 2009/10	0.74	(0.63 – 0.86)^***^	0.756	(0.68 – 0.85)^***^	1.55	(1.38 – 1.74)^***^	0.68	(0.61 – 0.76)^***^
OR for the trend	0.97	(0.96 – 0.99)^***^	0.98	(0.97 – 0.99)^***^	1.03	(1.02 – 1.04)^***^	0.97	(0.96 – 0.97)^***^

**Table 4:** Fulfilling the physical activity recommendation, physical and sporting activity of boys, temporal trends (odds ratio, 95 % confidence interval) over the HBSC survey cycles 2009/10 (n = 2,364), 2013/14 (n = 2,967), 2017/18 (n = 2,021), and 2022 (n = 3,074), controlled for age Source: HBSC Germany 2009/10, 2013/14, 2017/18, 2022

Boys	Physical activity recommendation fulfilled^1^ (n = 10,250)		High physical activity^2^ (n = 10,250)		Low physical activity^3^ (n = 10,250)		Sporting activity (≥4 days per week) (n = 10,134)	
	OR	(95 % CI)	OR	(95 % CI)	OR	(95 % CI)	OR	(95 % CI)
**Date of survey**								
2013/14 vs. 2009/10	0.98	(0.85 – 1.12)	0.87	(0.78 – 0.97)^*^	1.20	(1.05 – 1.38)^**^	1.05	(0.94 – 1.17)
2017/18 vs. 2009/10	0.83	(0.71 – 0.96)^*^	0.84	(0.75 – 0.95)^**^	1.18	(1.02 – 1.36)^*^	0.92	(0.82 – 1.04)
2022 vs. 2009/10	1.07 1.00	(0.94 – 1.22)	0.98	(0.88 – 1.09)	1.16	(1.01 – 1.33)^*^	0.88	(0.79 – 0.98)^*^
OR for the trend		(0.99 – 1.01)	1.00	(0.99 – 1.01)	1.01	(0.998 – 1.02)	0.99	(0.98 – 0.995)^**^

OR = Odds ratio, CI = confidence interval

^*^ significant p < 0,05

^**^ very significant p < 0,01

^***^ highly significant p < 0,001

^1^ Physical activity recommendation fulfilled = 60 minutes of physical activity seven days a week

^2^ High physical activity = 60 minutes of physical activity on five to seven days a week

^3^ Low physical activity = 60 minutes of physical activity on less than three days per week
